# Identification of Novel Inhibitors of Dietary Lipid Absorption Using Zebrafish

**DOI:** 10.1371/journal.pone.0012386

**Published:** 2010-08-25

**Authors:** Justin D. Clifton, Edinson Lucumi, Michael C. Myers, Andrew Napper, Kotaro Hama, Steven A. Farber, Amos B. Smith, Donna M. Huryn, Scott L. Diamond, Michael Pack

**Affiliations:** 1 Department Medicine, University of Pennsylvania School of Medicine, Philadelphia, Pennsylvania, United States of America; 2 Penn Center for Molecular Discovery, Institute for Medicine and Engineering, University of Pennsylvania, Philadelphia, Pennsylvania, United States of America; 3 Department of Chemistry, University of Pennsylvania, Philadelphia, Pennsylvania, United States of America; 4 Department of Embryology, Carnegie Institution, Baltimore, Maryland, United States of America; 5 Department of Cell and Developmental Biology, University of Pennsylvania School of Medicine, Philadelphia, Pennsylvania, United States of America; Sun Yat-Sen University, China

## Abstract

Pharmacological inhibition of dietary lipid absorption induces favorable changes in serum lipoprotein levels in patients that are at risk for cardiovascular disease and is considered an adjuvant or alternative treatment with HMG-CoA reductase inhibitors (statins). Here we demonstrate the feasibility of identifying novel inhibitors of intestinal lipid absorption using the zebrafish system. A pilot screen of an unbiased chemical library identified novel compounds that inhibited processing of fluorescent lipid analogues in live zebrafish larvae. Secondary assays identified those compounds suitable for testing in mammals and provided insight into mechanism of action, which for several compounds could be distinguished from ezetimibe, a drug used to inhibit cholesterol absorption in humans that broadly inhibited lipid absorption in zebrafish larvae. These findings support the utility of zebrafish screening assays to identify novel compounds that target complex physiological processes.

## Introduction

Inhibition of dietary lipid absorption is an evolving strategy to treat cardiovascular complications of disorders of lipid metabolism. Two commonly used drugs in this class of pharmacological agents, orlistat and ezetimibe improve the serum lipoprotein profiles of patients that are at high risk for acute coronary syndrome, stroke and sudden death, and therefore may be used as an adjuvant or alternative to HMG co-reductase inhibitors (statins) for the primary and secondary prevention of these disorders [1]–[5]. Although confirmation of the efficacy of this pharmacological approach awaits completion of large clinical trials, the adjuvant use of these compounds is common in patients that do not meet targeted reductions of lipoproteins while taking statins [6]–[10].

Given the high prevalence of lipid metabolism disorders it is desirable to identify lead compounds that can be developed into new drugs that inhibit lipid absorption via novel mechanisms. Here we report the utility of using the zebrafish for this purpose. Because of their small size, optical transparency zebrafish larvae are well suited for chemical library screens using fluorescent, histochemical or morphological assays [11]–[16]. Indeed, a great advantage of chemical screens in zebrafish is the ability to rapidly assess compound efficacy and toxicity in vivo. Given the high degree of conservation of lipid metabolism in teleost fish and mammals [17]–[20], it is likely that compounds identified in a zebrafish screen will act through comparable mechanisms in mammals.

Here we report the results of a pilot screen of a non-biased chemical library through which we identified 7 novel compounds that inhibited the absorption of fluorescent lipid analogues. We show that compounds identified in the primary screening assay can be rapidly prioritized for testing in mammals using a variety of simple, yet highly informative in vivo secondary assays. The secondary assays also provided insights into the compounds' mechanism of action, which could be distinguished from the effects of orlistat and ezetimibe in zebrafish larvae. Surprisingly, we found that ezetimibe inhibited absorption of not only cholesterol analog, but also long chain fatty acid and phopholipid analogs. Together, these findings demonstrate the feasibility of conducting screens for compounds that interfere with complex physiological processes using the zebrafish.

## Results

The screening assays used for this study were derived from previous work using fluorescent lipid reporters in zebrafish larvae [19]. Following their ingestion, the fluorescent metabolites of these reporters are first detected in the gallbladder of live larvae and later the intestinal lumen following gallbladder contraction. The compounds are used at low concentrations and they are rapidly absorbed from the intestinal lumen, thus their fluorescence emission is not detected in the intestinal lumen immediately after ingestion or when absorption in inhibited. Fluorescence emission from one of the analogues, the phospholipid PED-6, is quenched prior to metabolism by luminal phospholipase. Thin layer chromatographic analyses of bile from adult fish, or total body lipids of 5 dpf larvae, showed that PED-6, which is labeled with a BODIPY labeled short chain fatty acid (C5) at the sn-2 position, is metabolized to cholesterol esters, phospholipids and possibly, triglyceride (19 and data not shown). Free PED6 was not detected in either assay.

**Figure 1:**
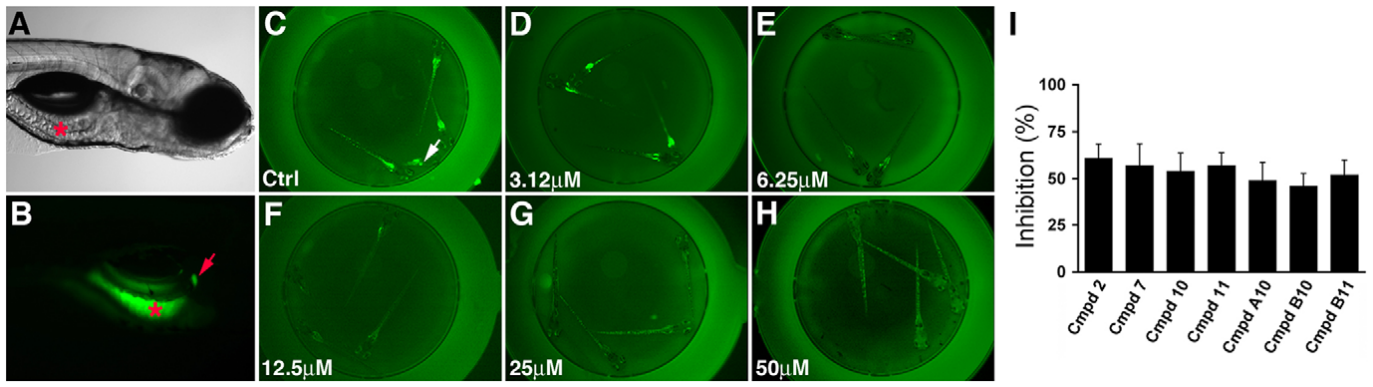
Chemical screen identifies novel inhibitors of zebrafish lipid metabolism. (A, B) Bright field and fluorescent images of a 6 day post-fertilization wild type zebrafish larva that had ingested the PED6 lipid reporter. Fluorescence is detected in the intestine (asterisk) and gallbladder (arrow). (C–H) Representative dose response assay shows inhibition of PED6 metabolism in larvae treated with compound 2. Inhibition of PED6 metabolism is first detected at 6.25 uM. Larvae (6 day post-fertilization) are arrayed in a 96-well plate. (I) Quantification of the effect of active compounds (25 uM) on PED6 metabolism, as determined by total gallbladder and intestinal fluorescence measured in individual larvae. Error bars – standard deviation.

For the primary screen, 5 day post-fertilization larvae were arrayed in 96 well plates and soaked overnight in test compounds (25 uM in 2% DMSO). The following morning larvae were soaked in PED-6 for 6 hours (Figure 1A, B) after which a qualitative visual assessment of gallbladder fluorescence was made using an inverted compound microscope. Reduced gallbladder fluorescence, the endpoint we use to identify active compounds in the primary screen, could not differentiate compounds that inhibited lipid absorption from those that interfered with swallowing, phospholipase activity or hepatic metabolism and biliary secretion. As described below, secondary assays were devised to distinguish these mechanistic possibilities.

Preliminary results of a pilot screen of 3,840 compounds from the MLSCN library (http://mli.nih.gov/mli/) have been reported (http://pubchem.ncbi.nlm.nih.gov/assay/assay.cgi?aid=686&loc=ea_ras; http://pubchem.ncbi.nlm.nih.gov/assay/assay.cgi?aid=691&loc=ea_ras). Here were identify three additional compounds recovered in this screen and provide a detailed account of the screening assay and the results of newly devised secondary assays designed to define mechanism of action and prioritize compounds for testing in mammalian models.

Larvae tolerated overnight incubation in the majority of the 3,840 compounds analyzed in the primary screen, however 67 compounds (1.75%) caused larval death or severely compromised cardiac circulation and were therefore deemed toxic. 50 compounds (1.3%) caused either complete or partial inhibition gallbladder fluorescence. When re-tested in a qualitative visual assay of PED-6 metabolism, 15 of these compounds were considered active in a dose responsive fashion (0.3% of the total number screened). 12 of the 15 compounds identified in the primary screen were tested in adult fish; 5 compounds were deemed active based on reduced gallbladder fluorescence derived from PED-6 (not shown) while 7 were either inactive and or toxic in adult fish and not studied further. Together with the 3 compounds that were not available in sufficient quantity to be tested in adult fish, this left 8 compounds for testing in secondary assays (Table S1).

The visual dose response assays conducted in larvae (n = 5) arrayed in the 96 well plates showed that 2 of the 8 compounds first inhibited PED-6 processing at 6.25 uM (compounds 2 and 10), whereas the remaining compounds were first active at 25 uM (Figure 1C–H shows dose response for compound 2). In separate experiments (described in the Methods section), combined gallbladder and intestinal fluorescence of individual compound treated larvae was quantified using fluorescence microscopy. This showed that the active compounds reduced PED-6 metabolism between 51%–67% (Figure 1I and Figure S1). Of the 8 active compounds, only 1 has been used in humans; clofazimine (compound 10), a rhiminophenazine dye with antimicrobial and anti-inflammatory activity used to treat leprosy and other types of mycobacterial infections [21]. Although intestinal toxicity has been reported with long term use of high doses of this drug, no prior reports of altered lipid absorption have been reported [22].

We devised a series of secondary assays that allowed us to further characterize the active compounds' mechanism of action and prioritize them for testing in mammals.

We assayed the effect of the active compounds on the ingestion of fluorescent microspheres to control for the possibility that they prevented swallowing of PED-6 from the larvae's aqueous media. This assay confirmed normal swallowing in 7 of 8 active compounds. Interestingly, the 1 compound that inhibited swallowing (compound 1; Figure S2) had no obvious effect on larval motility or cardiac function.

We assayed the effect of the active compounds on the metabolism of fluorescent cholesterol and fatty acid analogues because these dietary lipids are differentially absorbed and or processed by enterocytes compared with the phospholipid used for the primary screen, PED6.

**Figure 2:**
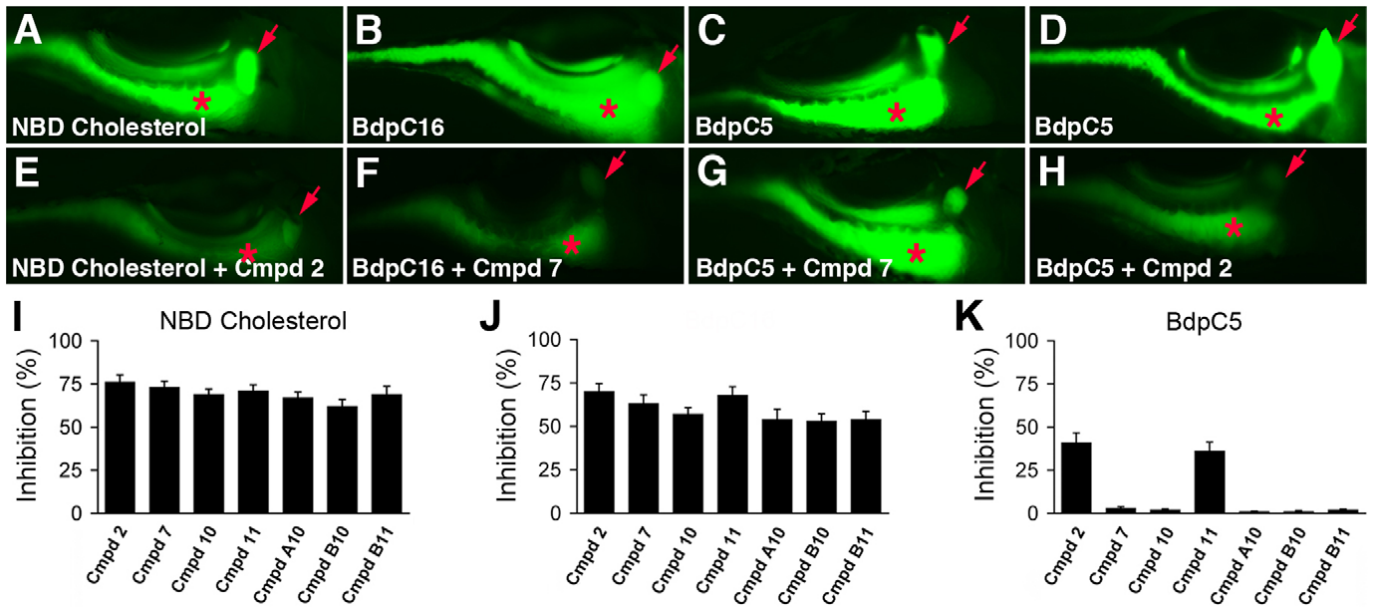
Cholesterol and fatty acid metabolism in zebrafish larvae treated with novel lipid absorption inhibitors. (A–H) Representative fluorescent images of live 6 day post-fertilization wild type and compound treated larvae following ingestion of NBD cholesterol (A, E), the LCFA BdpC16 (B, F), and the SCFA BdpC5 (C, D, G, H). All compounds used at 25 uM. Larvae treated with compound 2 showed reduced metabolism of NBD-cholesterol, BdpC16 (not shown), and BdpC5 whereas larvae treated with compound 7 have reduced metabolism of BdpC16, but not BdpC5. (I, J, K) Effect of the active compounds on lipid reporter metabolism, as determined by gallbladder and intestinal fluorescence measured from individual larvae. Red arrow - gallbladder; red asterisk - intestine. Error bars – standard deviation.

Recent studies have shown that the intestinal absorption of dietary cholesterol is dependent on the Neiman Pick Type C 1-Like 1 protein [NPC1L1; 23, 24]. Although the function of NPC1L1 is still debated, it is generally agreed upon that it as a cholesterol transporter embedded within the apical enterocyte membrane [25]–[27]. NPC1L1 has not been implicated in phospholipid absorption, thus it was not predicted that the screen compounds, which were identified by their inhibition of phospholipid (PED-6) absorption, would interfere with absorption of a fluorescent cholesterol analog, NBD-cholesterol. Surprisingly, each of the 7 active compounds inhibited metabolism of NBD-cholesterol, as determined by levels of biliary and intestinal fluorescence (Figure 2A, E, I and Figure S1).

We next measured the effect of the active compounds on the absorption of fluorescent short chain fatty acid (SCFA) and long chain fatty acid (LCFA) analogs. (Figure 2 and Figure S1). The distinction between acyl-chain length is important because LCFA are thought to be taken up from the intestinal lumen by a protein mediated process whereas as SCFA are thought to enter the enterocytes via simple diffusion [28], [29]. In addition, LCFA require incorporation into lipoprotein particles for transport from enterocytes to the liver whereas SCFA enter the blood directly and are transported bound to albumin and other serum proteins [29]. All 7 compounds inhibited metabolism of the LCFA C-16 bodipy (Figure 2B, F, J) but only 2 had an effect on SCFA C-5 bodipy metabolism (Figure 2C, G, D, H, K). Inhibition of native C5-bodipy processing by compounds 2 and 11 (Figure 2K) was less pronounced than inhibition of processing of LCFA, NBD-cholesterol or PED6 (Figure 2J, I; 1I).

**Figure 3:**
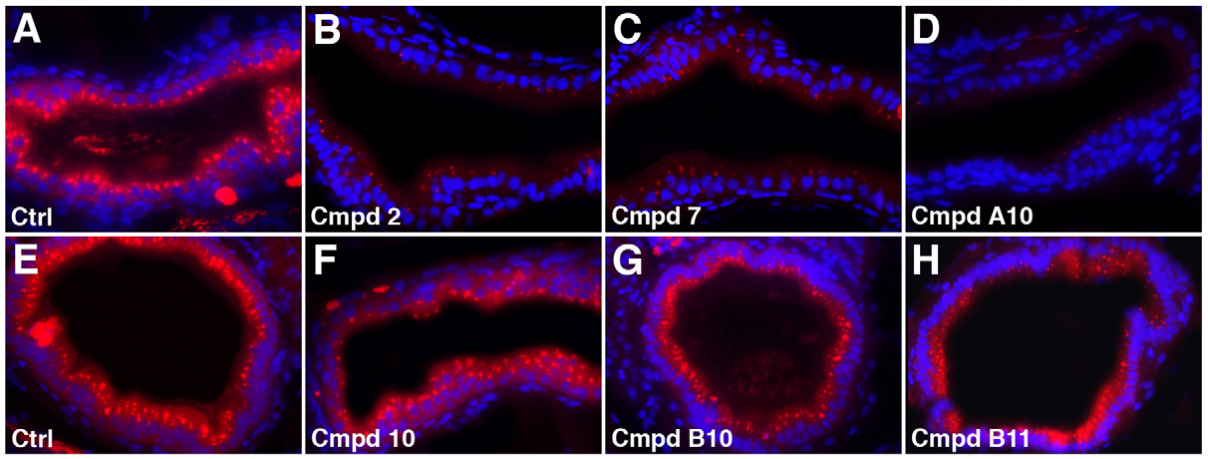
Endocytosis in zebrafish larvae treated with novel lipid absorption inhibitors. (A–H) Histological cross-sections through the anterior intestine of control and compound treated larvae following ingestion of AM1-43. AM1-43 uptake is inhibited in larvae treated with compounds 2, 7 and A10 whereas it is only slightly diminished in larvae treated with compounds 10, B10 and B11. Cell nuclei (blue) stained with Dapi. All compounds used at 25 uM.

Each of the active compounds from the primary screen inhibited PED6, NBD-cholesterol and Bodipy-C16 (LCFA) metabolism. In contrast, orlistat, a pancreatic lipase inhibitor, and ezetimibe, which targets NPC1L1, are reported to inhibit absorption of only dietary 1 lipid class; triglycerides, and cholesterol and structurally related phytosterols, respectively. To determine whether the non-selectivity of the active compounds arose from a non-specific disruption of endocytic absorptive pathways in enterocytes, we assayed in vivo processing of the styryl dye AM1-43. AM1-43 is a fixable derivative of FM1-43, a reagent that has been extensively used to study endocytosis [30]. When ingested by zebrafish larvae, AM1-43 strongly labels the apical plasma membrane of enterocytes. The number and size of AM1-43 labeled vesicles that can be detected in the cytoplasm of these cells provides a qualitative assessment of bulk endocytosis through the apical plasma membrane [31]. 3 of the 7 active compounds (compounds 2, 7, A10) caused a marked reduction in AM1-43 processing (Figure 3A–D). Fluorescent cytoplasmic vesicles could only be detected in small percentage of the enterocytes from these larvae (n  =  a minimum of 10 sections from 7 compound treated and wild type larvae). The vesicles that were detected were also smaller and had lower fluorescent emission. The effect of the remaining 4 compounds (10, 11, B10, B11; Figure 3E–H and data not shown) was deemed less pronounced because a larger number of fluorescent vesicles were detected in enterocytes of treated larvae.

**Figure 4:**
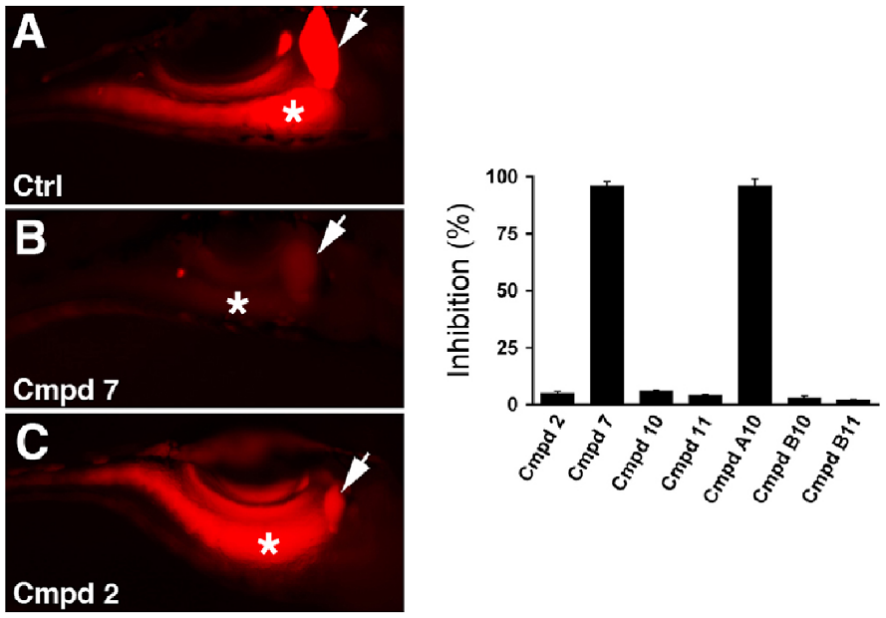
Digestive protease activity in compound treated larvae. (A–C) Representative fluorescent images of live wild type and compound treated larvae following ingestion of a quenched bodipy conjugate of casein. Bright fluorescence is seen in the intestine of the wild type larva and the larva treated with compound 2, while minimal fluorescence is detected in the intestine of the larva treated with compound 7. (D) Quantification of the effect of the active compounds on bodipy-casein metabolism, as determined by gall bladder and intestinal fluorescence measured from individual larvae. Arrow - gallbladder; asterisk - intestine. Error bars – standard deviation. All compounds used at 25 uM.

To determine whether the active compounds identified in the primary screen affected other aspects of digestive physiology we assayed protease activity using a quenched bodipy labeled casein protein. Cleavage of this reporter by pancreatic proteases generates fluorescent peptides that can be detected in the intestinal lumen of wild type larvae [32]. Intestinal fluorescence derived from the casein reporter was minimally reduced in larvae treated with 5 of 7 compounds (compounds 2, 10, 11, B10, B11; Figure 4). Treatment with 2 compounds (7 and compound A10) caused a profound reduction in the metabolism of the casein reporter (Figure 4).

**Figure 5:**
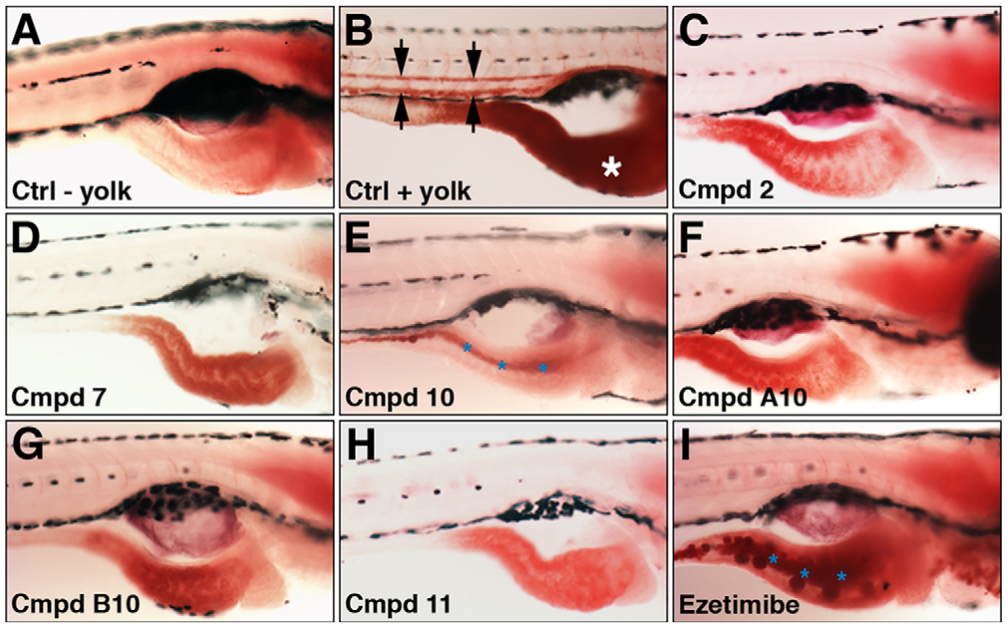
Reduced intestinal lipid absorption in ezetimibe and compound treated zebrafish larvae. (A–L) Lateral views of 6 day post-fertilization larvae following fixation and staining with oil red o to mark neutral lipid. (A) Unfed wild type larva. (B) Wild type larva fed 1% egg yolk. Arrows point to lipid in vasculature. (C–I) Compound treated larvae (25 uM) fed 1% egg yolk. There is reduced intestinal lipid in the compound treated larvae, with no evidence of lipid in the vasculature. Hepatic lipid accumulation is not detected. Lipid is present in the intestinal lumen of larvae treated with compound 10 and ezetimibe (50 uM; blue asterisk).

Changes in gallbladder and intestinal fluorescence detected in the primary screening assay detected could have arisen from a reduction in either intestinal and or hepatic lipid processing. We fed compound treated larvae egg yolk and after allowing time for its absorption, we performed whole mount stainings using the lipophilic dye oil red o (ORO) to determine whether yolk-derived lipids accumulated in either organ (Figure 5). Wild type larvae fed egg yolk had strong ORO staining of the anterior intestine, as well as the blood stream, the latter arising from lipid in circulating lipoproteins [17] (Figure 5A, B). Manual dissection of the intestine showed that the ORO staining derived from small lipid droplets within the enterocyte cytoplasm (data not shown). Lipid within the intestinal lumen was not detected in any wild type larvae (n = 10). Each of the 7 active compounds tested reduced intestinal lipid (Figure 5C–H and data not shown). Lipid was detected in enterocytes of all compound treated larvae, but at far lower levels than in wild type, except in larvae treated with compound 10 (Figure 5E). Here luminal lipid was detected. No evidence of hepatic lipid accumulation was evident. Collectively, these findings are compatible with reduced intestinal lipid absorption in compound treated larvae.

Compound synergy was examined in binary combinations of the 7 remaining active compounds with each other and with ezetimibe. Each compound was assayed at the highest concentration deemed inactive and the lowest dose considered active in the visual dose response experiment. These experiments identified potential synergism between compounds 2 and 10 (data not shown).

The two most commonly prescribed lipid absorption inhibitors, orlistat and ezetimibe, are generally considered to be selective inhibitors of triglyceride, and cholesterol and phytosterol absorption, respectively. To gain a better understanding of the mechanism of action of the novel compounds identified in our screen, we examined how these drugs affected absorption of fluorescent lipid reporters in zebrafish larvae.

**Figure 6:**
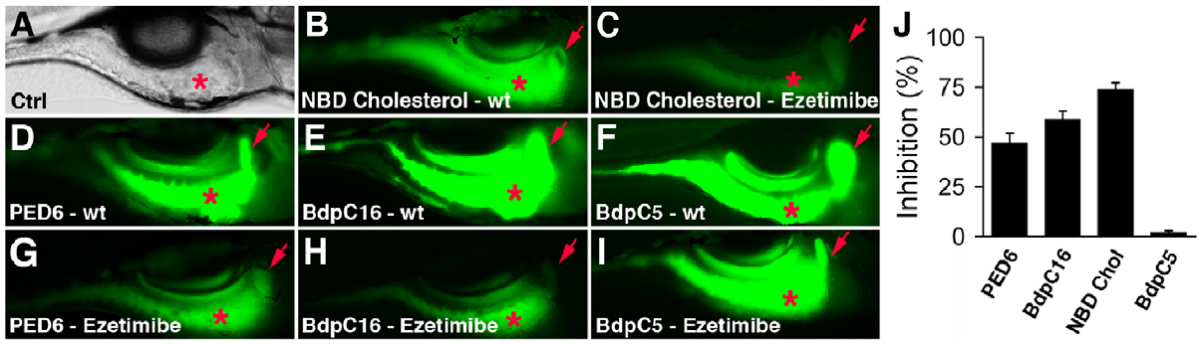
Ezetimibe inhibits lipid metabolism in zebrafish larvae. (A) Bright field image of a 6 day post-fertilization wild type (wt) zebrafish larva, lateral view. Red asterisk - intestine. (B–I) Gallbladder (red arrow) and intestinal (red asterisk) fluorescence in live 6 day post-fertilization wt and ezetimibe (50 uM) treated larvae following ingestion of fluorescent lipid reporters. (B, C) NBD-cholesterol; (D, G) PED6, (E, H) C16 bodipy (BdpC16), and (F, I) C5 bodipy (BdpC5). (J) Mean reduction in lipid reporter metabolism in ezetimibe vs wild type larvae. Error bars – standard deviation.

Both drugs were assayed in an identical fashion as the screen compounds. Orlistat had no effect on the metabolism of any of the lipid reporters (data not shown). This was predicted, however because none are processed by pancreatic lipase, which is responsible for hydrolysis of triglycerides [33]. In contrast to orlistat, ezetimibe was predicted to inhibit absorption of NBD-cholesterol because the amino acid domain of dog Npc1l1 required for high affinity binding to ezetimibe [34], [35] is highly conserved in both human NPC1L1 and the predicted zebrafish Npc1l1 protein (42 identical, 11 conserved and 10 non-conserved residues; Figure S3). Indeed, larvae treated overnight with the highest ezetimibe dose tested (50 uM) showed a 78% reduction of gallbladder and intestinal fluorescence derived from NBD-cholesterol (Figure 6A–C, J). Treatment with lower doses (37.5 uM, 25 uM, 12.5 uM, 6.25 uM) showed proportionately less inhibition (Figure S4). Unexpectedly, ezetimibe also reduced metabolism of the phospholipid PED-6 and the saturated long chain fatty acid (LCFA) Bodipy-C16 (Figure 6D, E, G, H, J). As predicted, ezetimibe had minimal effect on the metabolism of SCFA Bodipy-C5 (Figure 6F, I, J). ORO stainings of yolk-fed larvae confirmed reduced lipid absorption was reduced by ezetimibe treatment (Figure 5I). Ezetimibe had no effect on digestive protease function in zebrafish larvae (data not shown).

**Figure 7:**
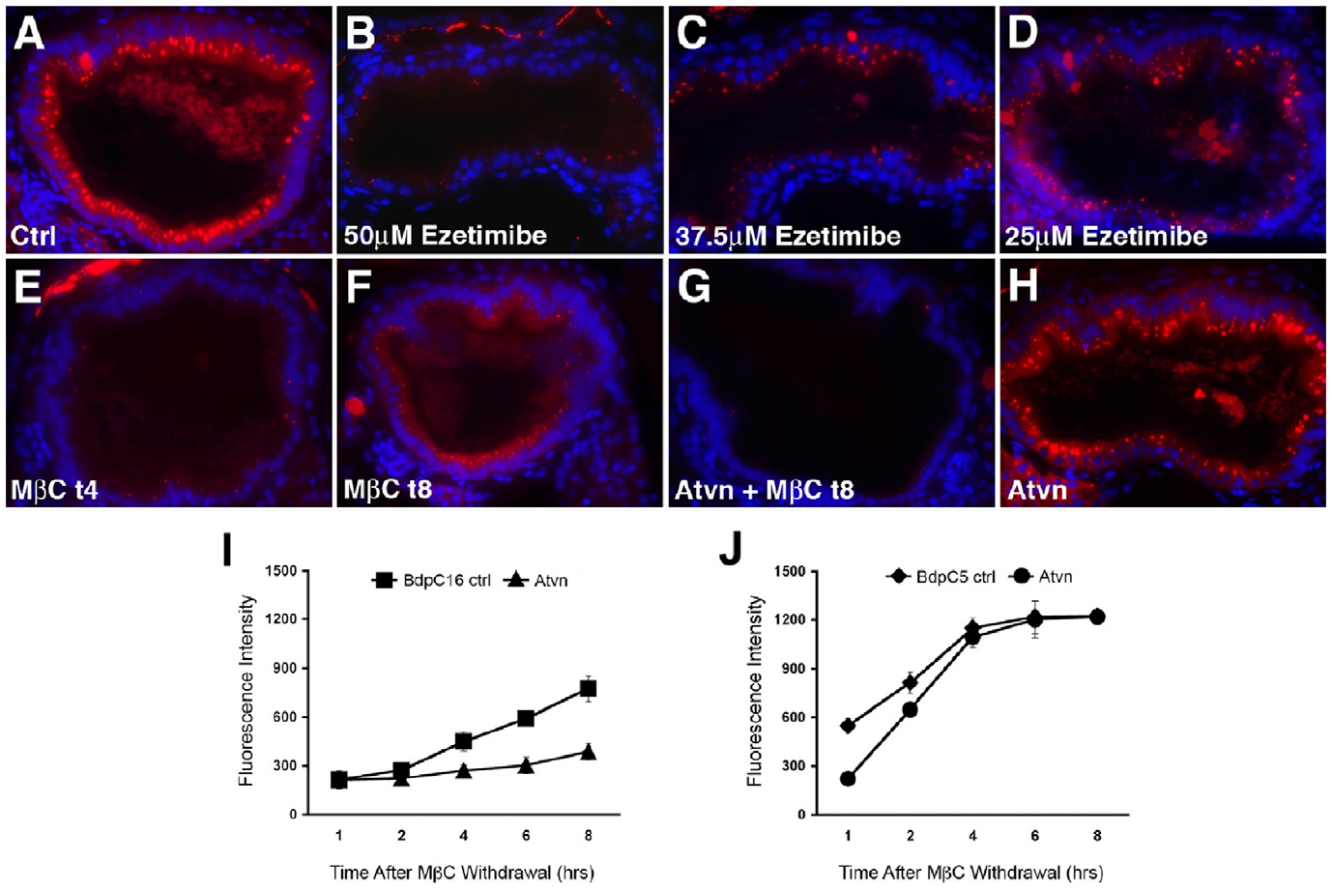
Membrane cholesterol depletion and ezetimibe inhibit endocytosis and fatty acid metabolism in zebrafish larvae. (A–H) Histological cross sections through the anterior intestine of 6 day post-fertilization zebrafish larvae following ingestion and processing of the styryl dye AM1-43. (A) Red fluorescent endocytic vesicles are evident in enterocytes of a wild type larva soaked in AM1-43 for 4 hours. (B–D) Ezetimibe (50 uM) strongly inhibits AM1-43 uptake by enterocytes in a dose dependent manner. (E, F) A larva treated with methyl-β-cyclo-dextrin (MβC) shows little AM1-43 uptake 4 hours after MβC withdrawal (MβC t4) whereas AM1-43 uptake can be detected at 8 hours after MβC withdra25al (MβC t8). (G) Concomitant inhibition of cholesterol synthesis with atorvastatin (Atvn) prevents recovery of AM1-43 uptake following MβC withdrawal. (H) Atvn alone has no effect on AM1-43 uptake. (I) Metabolism of the LCFA BdpC16 (I) but not SCFA BdpC5 (J), as measured by gallbladder and intestinal fluorescence, is inhibited by membrane cholesterol depletion caused by MβC. (I) BdpC16 metabolism recovers following MβC withdrawal but not in the presence of Atvn. (J) By contrast, recovery of BdpC5 metabolism from the effects of MβC that are independent of cholesterol depletion occurs with or without Atvn treatment.

Previous work suggests that ezetimibe interferes with intestinal cholesterol absorption by disrupting the incorporation of NPC1L1 into clathrin-coated vesicles [27]. This mechanism does not predict that ezetimibe will interfere with fatty acid or phospholipids uptake by enterocytes, neither of which are known to be dependent on NPC1L1. Because of this, we speculated that ezetimibe had a broader disruptive effect on intestinal endocytic mechanisms. To examine this, we measured uptake of AM1-43 in ezetimibe treated larvae. Compared with control larvae, ezetimibe (50 uM) treated larvae had a markedly reduced number of AM1-43 labeled vesicles in enterocytes of the anterior intestine, the site of lipid absorption in zebrafish larvae (Figure 7A, B). The effect of ezetimibe on AM1-43 uptake was dose responsive (Figure 7C, D).

To gain additional insight into the mechanism of action of ezetimibe as well as the active compounds that affected endocytosis (2, 7, A10), we compared their effect on AM1-43 metabolism with the effect of methyl-β-cyclodextrn (MβC), a reagent that disrupts membrane lipid rafts by extracting membrane cholesterol [36]–[38]. Pretreatment of zebrafish larvae with MβC for four hours strongly inhibited endocytic uptake of AM1-43 by enterocytes (Figure 7E). Recovery of endocytic function was detected eight hours after MβC withdrawal (Figure 7F), but was prevented in larvae unable to replenish membrane cholesterol because of concomitant treatment with the cholesterol synthesis inhibitor atorvastatin (Figure 7G). Atorvastatin treatment on its own had no effect on AM1-43 processing (Figure 7H). Like ezetimibe and the compounds that interfered with AM1-43 processing, MβC inhibited C-16 bodipy metabolism, and this too was reversed by repletion of membrane cholesterol (Figure 7I). MβC had minimal effect on C-5 bodipy metabolism (Figure 7IJ), most likely because enterocytes absorb SCFA via passive diffusion.

## Discussion

The principal findings of this study support the utility of zebrafish screening assays for lead compounds that can be developed into new drugs that inhibit lipid absorption. The screen utilized fluorescent lipid analogs to directly assay intestinal lipid absorption in larvae treated with novel chemical compounds, thus distinguishing it from a study that examined the effects of known drugs on endogenous yolk-lipid metabolism in younger zebrafish larvae [39]. Using this screen we show that it is not only possible to rapidly identify compounds that disrupt lipid metabolism with comparable efficacy to ezetimibe, the most commonly used drug in this class of pharmaceutical agents, but importantly, that secondary assays allow their prioritization for subsequent evaluation in mammalian models. Thus, even though a relatively high percentage (1.3%) of the compounds analyzed in our primary screen were initially scored as active, most of these were quickly determined to be either false positives (1.1%), or were acutely toxic to adult fish (0.1%). Of the remaining 8 compounds (0.2%), 1 was shown to inhibit swallowing, thus leaving 7 compounds for more detailed secondary analyses.

The secondary assays we devised took advantage of the ability to conduct simple studies in zebrafish larvae that have well formed organ systems with remarkably conserved physiology. The first set of assays evaluated how each of the active compounds affected metabolism of 3 different classes of lipids. 5 of the 7 compounds studied functioned comparably to ezetimibe, which inhibited processing of cholesterol, LCFA and phospholipids analogues. The remaining 2 compounds inhibited processing of these lipids as well as the SCFA C-5 bodipy whose absorption was unchanged even following membrane disruption with MβC. Irrespective of the cellular processes affected by these 2 compounds, the fact that they inhibited uptake of a lipid (SCFA) that normally enters enterocytes via simple diffusion warrants their elimination from consideration for drug development.

The second secondary assay scored each compounds' effect on enterocyte endocytic pathways, as measure by AM1-43 processing. Although not as easily quantified as fluorescent lipid metabolism, the number of enterocyte fluorescent endocytic vesicles was significantly reduced in larvae treated with 3 of the active compounds. Although the effects of these 3 compounds were comparable to ezetimibe, additional secondary assays eliminated them from further consideration for drug testing. One of the three compounds (compound 2) inhibited SCFA (Bodipy-C5) metabolism. The remaining two compounds (compound 7, A10) inhibited processing of a quenched fluorescent casein derivative that is normally metabolized by pancreatic proteases secreted in response to CCK [40], an intestinal hormone whose cognate receptor is metabolized in pancreatic acinar cells and other tissues by clathrin-dependent and clathrin-independent endocytic mechanisms. Given their effects on enterocyte membrane dynamics (as measured by AM1-43 processing), we speculate that compounds 7 and A10 interfered with CCK activity either by disrupting endocytosis of the CCK receptor (since this been reported to promote CCK signaling in pancreatic acinar cells; 40, 41), or by altering ligand induced changes in CCK receptor oligomerization or sequestration in the acinar cell plasma membrane [41], [42].

Based on the secondary assays, the remaining 3 active compounds (compounds 10, B10 and B11) could be considered candidates for testing in mammals. Because all of the compounds interfered with the absorption of phospholipid, cholesterol and LCFA reporters, we initially thought that they non-specifically interfered with enterocyte absorptive mechanisms, thus potentially precluding their utility for drug development. Arguing against this, we found that ezetimibe had comparable effects on lipid absorption in zebrafish larvae. While this could indicate that ezetimibe functions differently in zebrafish than in mammals, recent studies suggest that ezetimibe interferes with dietary fat absorption in mice and humans [43], [44]. As these effects on fat absorption were relatively modest, they may have been overlooked in previous studies. Nonetheless, they are consistent with our zebrafish data.

The comparable effects of ezetimibe and MβC on AM1-43 processing and fatty acid absorption lead us to speculate that ezetimibe has a broader effect on enterocyte membrane dynamics than previously recognized [27], [45]. As result, ezetimibe might interfere with the incorporation into lipid rafts of membrane proteins that are required for fat absorption, in addition to its effects on NPC1L1. A recent study suggests such a role for the SR-BI/CLA-1 scavenger receptor [46], which has previously implicated as playing a role in dietary fat absorption [47]–[49]. As the screen compounds we considered best suited for testing in mammals had a less pronounced effect on AM1-43 uptake than ezetimibe, it is conceivable that they inhibit lipid absorption via unique mechanisms.

## Materials and Methods

Zebrafish husbandry and care of embryos and larvae has been previously described [50]. All animals were handled in strict accordance with good animal practice as defined by the relevant national and/or local animal welfare bodies, and all animal work was approved by the institutional IACUC.

5 day post-fertilization zebrafish larvae (n = 6) were added to each well of a 96 well plate in 80 ul of E3 embryo media. To each well, 20 ul of 125 uM stock of one test compound in 2% DMSO was added (final concentration 25 uM). The larvae were incubated overnight at 28 degrees celcius. The following morning PED-6 (Invitrogen) was added to each well at a final concentration of 0.1 ug/ml. Gallbladder and intestinal fluorescence was determined by visual inspection of each well 6 hours later using a Olympus BX81 fluorescent inverted microscope. Compounds that caused a qualitative reduction in gallbladder and intestinal fluorescence were considered positive in the original primary screen. As not all larvae in the 96 well plates could be confidently scored, a compound was considered active if gallbladder fluorescence was reduced in at least 3 well visualized larvae. Active compounds identified in the primary screen were retested in a visual dose response assay. Most compounds were tested at 6.25 uM to 100 uM. Others were tested at 25 uM –100 uM. The dose response assay was conducted identically to the primary screen. Compounds were considered active if all larvae showed inhibition of gallbladder and intestinal fluorescence. For quantification of gallbladder and intestinal fluorescence individual larvae were removed from the 96 well plates and arrayed on a depression slide and imaged using an Olympus BX71 fluorescent microscope. Total gallbladder and intestinal fluorescence in digital images of each larva was quantified using Slidebook software.

Commercially acquired zetimibe tablets were crushed with a glass rod in a 10 mL round bottom flask, taken up into DMSO (4 mL), and stirred for 1 h at 23°C. The solution was filtered with an HPLC filter and water added (1 mL). The sample was purified utilizing preparative LC-MS and 8 mg of zetimibe was obtained. Zetimibe obtained employing these conditions was found to be analytically pure by LC-MS analysis. For all assays, 5 day post-fertilization zebrafish larvae were incubated overnight in purified ezitimibe at the test concentrations indicated and then subjected to assays as described for the compound treated larvae.

Assays of short chain fatty acid (SCFA; Bodipy-C5), long chain fatty acid (LCFA; Bodipy-C16) and cholesterol (NBD-cholesterol) were conducted identically to the primary screen as previously reported [19]. All reagents were purchased from Invitrogen. For the digestive protease assay, larvae were treated identically to the primary screen but instead of PED-6 the larvae were soaked in quenched bodipy-casein (EnzCheck- Invitrogen) as recently described [31]. For the swallowing assay, compound treated larvae were soaked in fluorescent microscpheres (In vitrogen) for 5 hours. The larvae were then washed and intestinal fluorescence quantified microscopically as previously noted. The AM1-43 assay was performed as previously described using larvae treated overnight with either the active compounds or ezetimibe [30]. Qualitative analysis of endocytosis was performed by examining enterocyte AM1-43 uptake in a minimum of 10 histological cross sections from 7 larvae within each experimental group. When indicated, larvae were incubated in methyl-β-cyclodextrin (Advasep-7; Biotium, Inc.) for 4 hours, washed for 2 hours and then soaked in AM1-43 with or without Atorvastatin (50 uM) as previously described [30]. Histological analyses were performed as previously reported [30].

Compounds 10, B10, and B11 were analyzed by LC/MS for purity and integrity using a Sunfire C18 5 mm column (4.6×50 mm) and a gradient elution system of CH3CN: H20 (10% to 90% over 10 minutes). Compound 10 was 100% pure by diode array detection, with molecular ion detected at 473.2 (M+1). Compound 13 was 100% pure by diode array detection, with molecular ion detected at 418.3 (M+1). Compound 14 was 88% pure by diode array detection, with molecular ion detected at 349.2 (M+1). The activity of each compound was confirmed in repeat assays using either the Bodipy-C16 or PED-6 lipid.

## Supporting Information

Table S1 Active compounds derived from primary and secondary screening assays.
(0.06 MB DOC)

Figure S1 Lipid reporter metabolism in ezetimibe and compound treated larvae: Values represent intestinal and gallbladder fluorescence. 4 larvae analyzed at each time point. Error bars are standard deviation from the mean of 3 independent experiments.
(2.27 MB DOC)

Figure S2 Swallowing assay in live zebrafish larvae: (A–F) Bright field and corresponding fluorescent images of control and representative compound treated 6 dpf zebrafish larvae following ingestion of fluorescent microscphreres. (G, H) Quantification of intestinal fluorescence in compound treated and sibling control larvae. Compound 1 strongly inhibits swallowing whereas swallowing is normal in larvae treated with compound 2. Each data point represents mean intestinal fluorescence of 6 larvae.
(0.32 MB DOC)

Figure S3 Conserved ezetimibe binding domain in human and zebrafish NPC1L1 protein: The 62 amino acid tract of NPC1L1 shown to mediate binding of ezetimibe (amino acids 510 to 572). 41 of the amino acids in the human and zebrafish proteins are identical, 11 are conserved and 10 and non-conserved. Conserved phenylalanine (F) and methionine (M) residues required for high-affinity binding are shown in red.
(0.02 MB DOC)

Figure S4 Ezetimibe inhibits cholesterol metabolism in zebafish larvae: Mean percent inhibition of intestinal and gallbladder fluorescence in ezetimibe treated larvae (6.25 uM, 12.5 uM, 25 uM and 37.5 uM). N = 6 larvae for each dose. Error bars indicate standard deviation.
(0.06 MB DOC)
