# Supplementary material for: Identification of Novel Inhibitors of Dietary Lipid Absorption Using Zebrafish
Source: PLoS One. 2010 Aug 25;5(8):e12386. doi: 10.1371/journal.pone.0012386 (PMC2928291; doi:10.1371/journal.pone.0012386)
Supplement: Table S1 — Active compounds derived from primary and secondary screening assays. (0.06 MB DOC) [file pone.0012386.s001.doc]

| **Cmpd** | **PubChem SID** | **Lowest Active Conc. Tested** | **Structure** |
| --- | --- | --- | --- |
| 1 | 858057 | 25 uM |  |
| 2 | 856867 | 6.25 uM |  |
| 7 | 860531 | 25 uM |  |
| 10 | 855829 | 6.25 uM |  |
| 11 | 4246938 | 25 uM |  |
| A10 |  | 25 uM |  |
| B10 |  | 25 uM |  |
| B11 |  | 25 uM |  |
