# Supplementary figures and images for: Identification of Novel Inhibitors of Dietary Lipid Absorption Using Zebrafish
Source: PLoS One. 2010 Aug 25;5(8):e12386. doi: 10.1371/journal.pone.0012386 (PMC2928291; doi:10.1371/journal.pone.0012386)

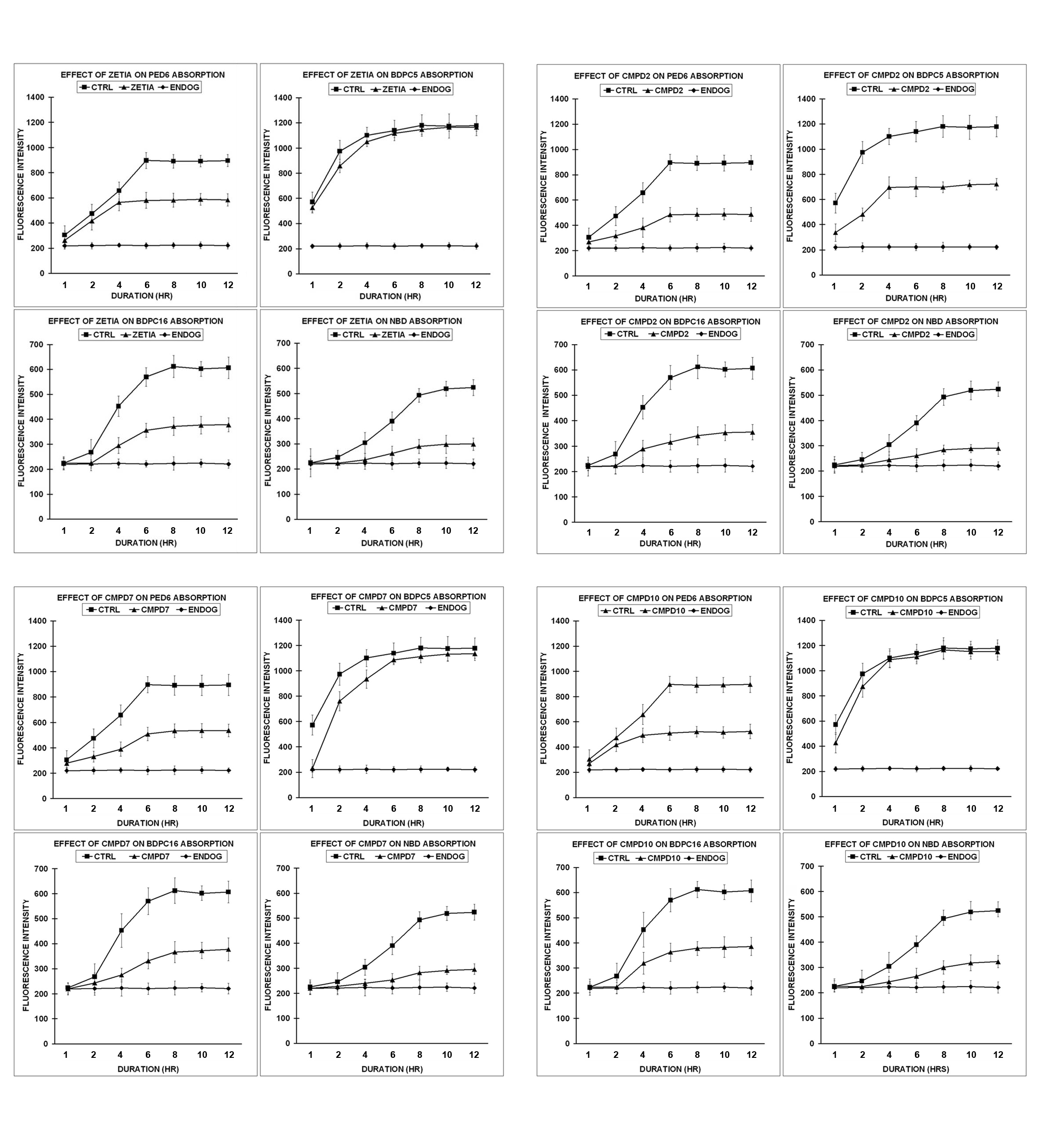


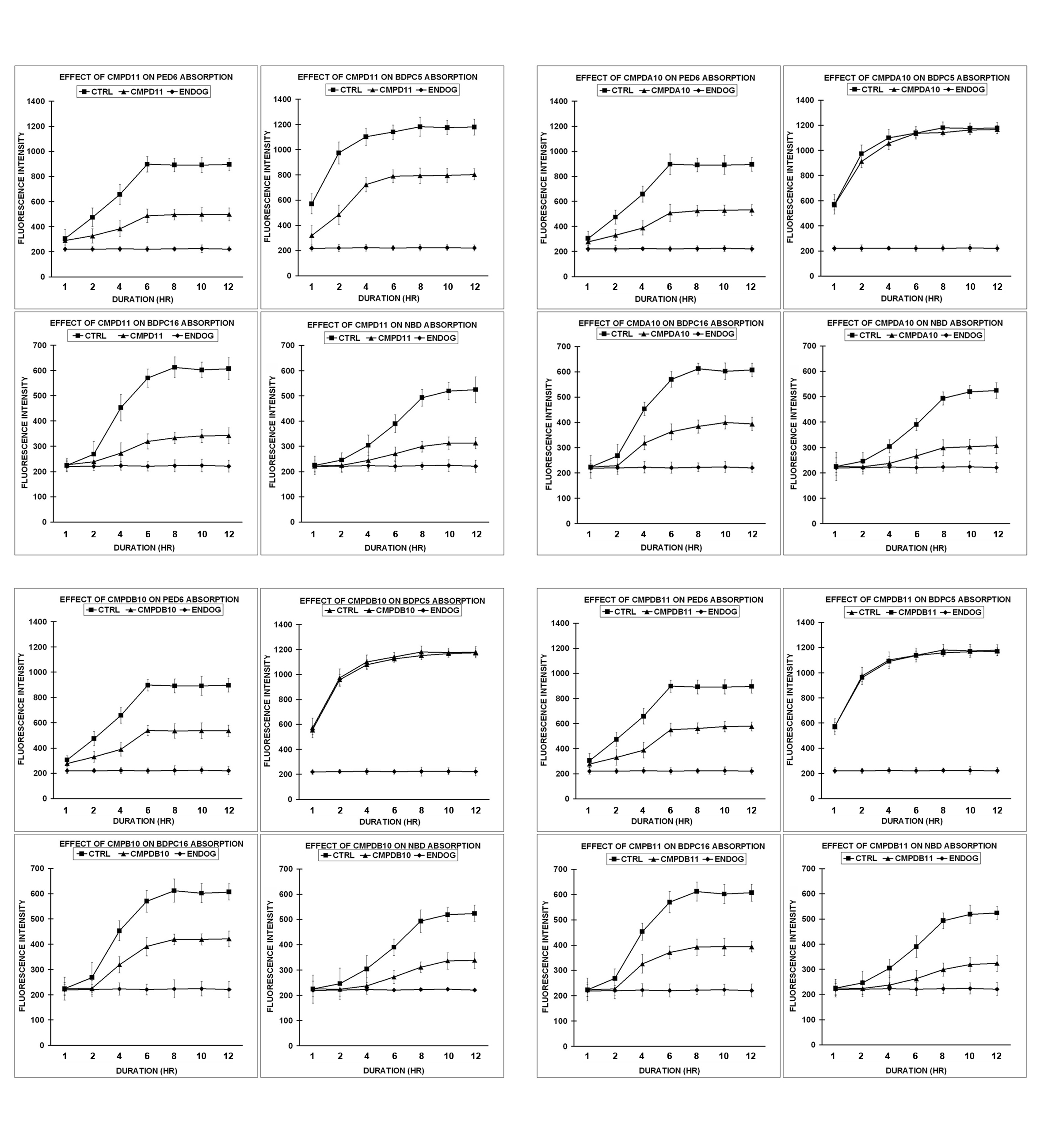

Supplement: Figure S1 — Lipid reporter metabolism in ezetimibe and compound treated larvae: Values represent intestinal and gallbladder fluorescence. 4 larvae analyzed at each time point. Error bars are standard deviation from the mean of 3 independent experiments. (2.27 MB DOC) [file pone.0012386.s002.doc]

**
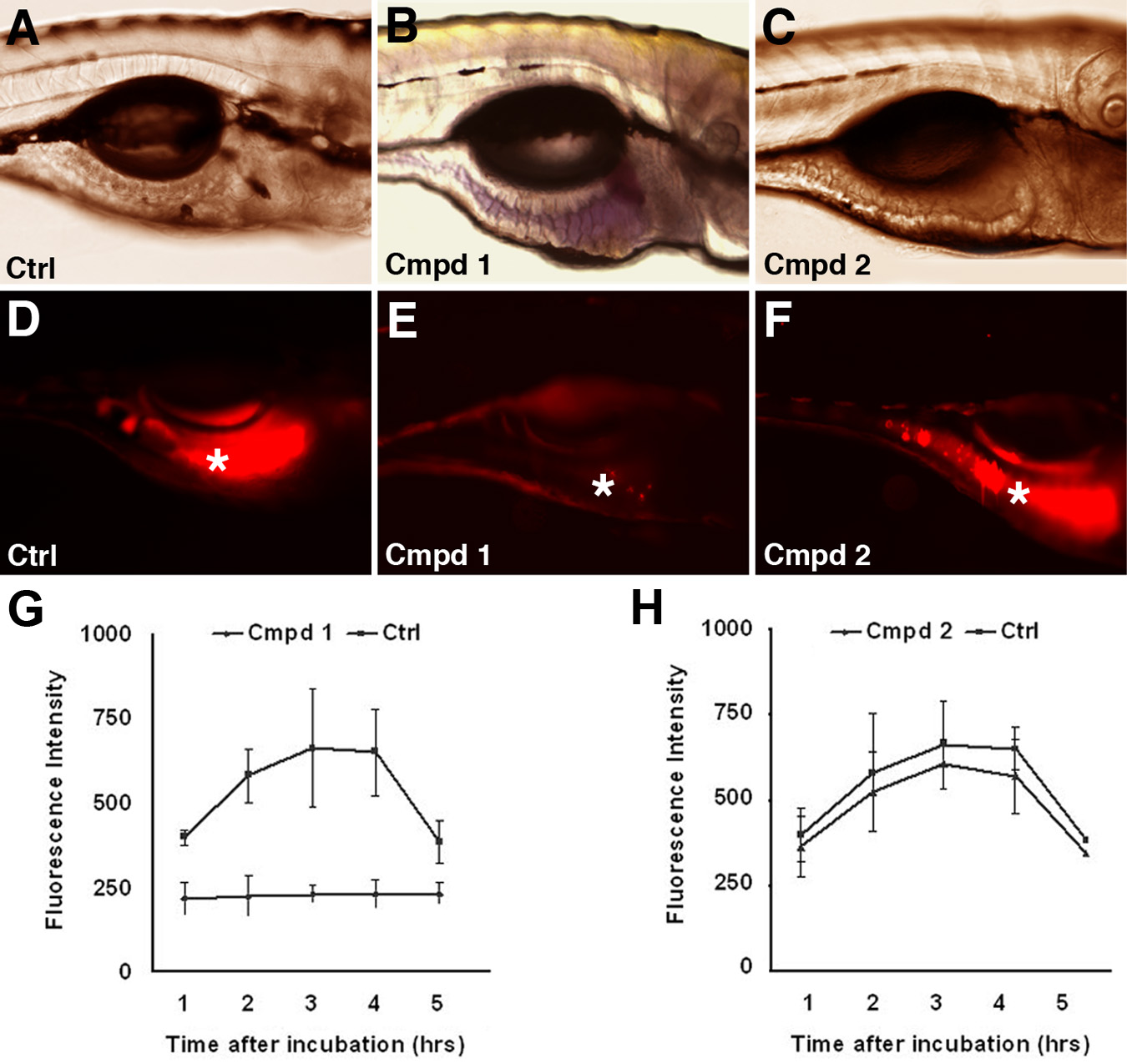
**

Supplement: Figure S2 — Swallowing assay in live zebrafish larvae: (A–F) Bright field and corresponding fluorescent images of control and representative compound treated 6 dpf zebrafish larvae following ingestion of fluorescent microscphreres. (G, H) Quantification of intestinal fluorescence in compound treated and sibling control larvae. Compound 1 strongly inhibits swallowing whereas swallowing is normal in larvae treated with compound 2. Each data point represents mean intestinal fluorescence of 6 larvae. (0.32 MB DOC) [file pone.0012386.s003.doc]

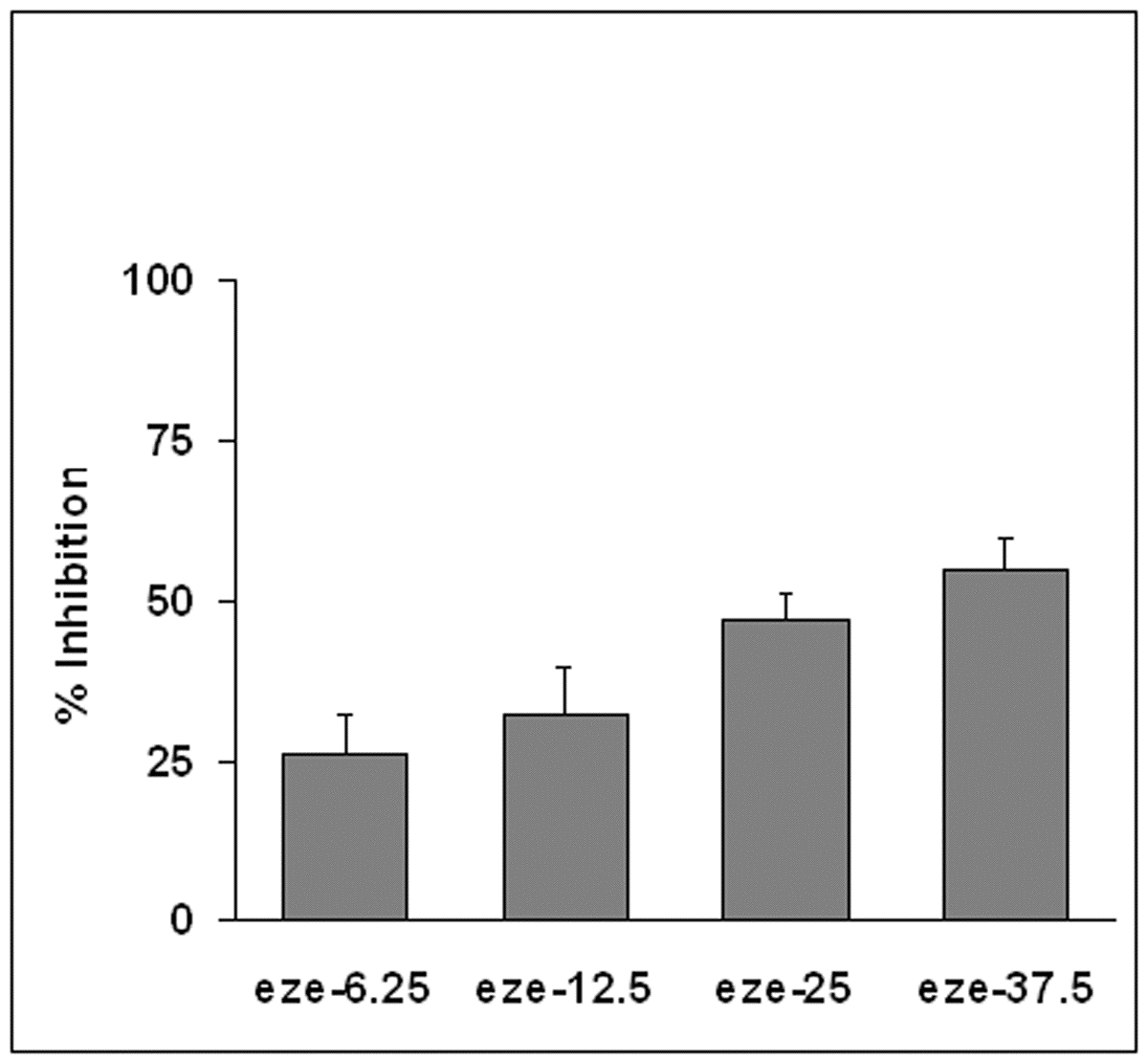

Supplement: Figure S4 — Ezetimibe inhibits cholesterol metabolism in zebafish larvae: Mean percent inhibition of intestinal and gallbladder fluorescence in ezetimibe treated larvae (6.25 uM, 12.5 uM, 25 uM and 37.5 uM). N = 6 larvae for each dose. Error bars indicate standard deviation. (0.06 MB DOC) [file pone.0012386.s005.doc]
