# Supplementary material for: Identification of Novel Inhibitors of Dietary Lipid Absorption Using Zebrafish
Source: PLoS One. 2010 Aug 25;5(8):e12386. doi: 10.1371/journal.pone.0012386 (PMC2928291; doi:10.1371/journal.pone.0012386)
Supplement: Figure S3 — Conserved ezetimibe binding domain in human and zebrafish NPC1L1 protein: The 62 amino acid tract of NPC1L1 shown to mediate binding of ezetimibe (amino acids 510 to 572). 41 of the amino acids in the human and zebrafish proteins are identical, 11 are conserved and 10 and non-conserved. Conserved phenylalanine (F) and methionine (M) residues required for high-affinity binding are shown in red. (0.02 MB DOC) [file pone.0012386.s004.doc]

**MGQTSQVDWKDHFLYCANAPLTFKDGTALALSCMADYGAPVFPFLAIGGYKGKDYSEAEALI Human**

**+G T +VDW+DHF+YC N+PL+FKD TAL +SCM+DYG PVFPFLA+GGY + Y+ AEALI**

**LGVTEKVDWRDHFIYCVNSPLSFKDITALGMSCMSDYGGPVFPFLAVGGYDNEQYTTAEALI Zfish**
